# Health Needs and Use of Services Among Children with Developmental Disabilities — United States, 2014–2018

**DOI:** 10.15585/mmwr.mm7112a3

**Published:** 2022-03-25

**Authors:** Mary E. Cogswell, Eric Coil, Lin H. Tian, Sarah C. Tinker, A. Blythe Ryerson, Matthew J. Maenner, Catherine E. Rice, Georgina Peacock

**Affiliations:** ^1^Division of Human Development and Disability, National Center on Birth Defects and Developmental Disabilities, CDC; ^2^Georgia State University, Atlanta, Georgia.

Developmental delays, disorders, or disabilities (DDs) manifest in infancy and childhood and can limit a person’s function throughout life* (*1*–*3*). To guide strategies to optimize health for U.S. children with DDs, CDC analyzed data from 44,299 participants in the 2014–2018 National Health Interview Survey (NHIS). Parents reported on 10 DDs,^†^ functional abilities, health needs, and use of services. Among the approximately one in six (17.3%) U.S. children and adolescents aged 3–17 years (hereafter children) with one or more DDs, 5.7% had limited ability to move or play, 4.7% needed help with personal care, 4.6% needed special equipment, and 2.4% received home health care, compared with ≤1% for each of these measures among children without DDs. Children with DDs were two to seven times as likely as those without DDs to have taken prescription medication for ≥3 months (41.6% versus 8.4%), seen a mental health professional (30.6% versus 4.5%), a medical specialist (26.0% versus 12.4%), or a special therapist, such as a physical, occupational, or speech therapist, (25.0% versus 4.5%) during the past year, and 18 times as likely to have received special education or early intervention services (EIS) (41.9% versus 2.4%). These percentages varied by type of disability and by sociodemographic subgroup. DDs are common, and children with DDs often need substantial health care and services. Policies and programs that promote early identification of children with developmental delays and facilitate increased access to intervention services can improve health and reduce the need for services later in life.^§^ Sociodemographic inequities merit further investigation to guide public health action and ensure early and equitable access to needed care and services.

The study included data from the 2014–2018 NHIS, an annual, multistage probability sample survey of the noninstitutionalized U.S. civilian population.^¶^ In-person interviews were conducted to obtain information on household members. Among families with children, a child questionnaire was administered to a knowledgeable adult (usually, and hereafter parent) about a randomly selected child (aged 0–17 years). During 2014–2018, the response rate for the child questionnaire was 59.2%–66.6%.

Parents of children aged ≥3 years were asked about their child’s functional abilities, health needs, and use of services (Supplementary Box 1, https://stacks.cdc.gov/view/cdc/115478) (*2*–*5*), as well as whether their child had any of 10 specific types of DDs (Supplementary Box 2, https://stacks.cdc.gov/view/cdc/115479). Children could be included in multiple diagnostic types of DDs; however, children with co-occurring learning disabilities and intellectual disabilities were excluded from the learning disability category. Weighted prevalence estimates of DDs and 95% CIs were calculated. The weighted percentages of children with each measure of reported functional ability, health needs, and use of specialty health care providers or education services were estimated overall, by selected sociodemographic characteristics, number of DDs, and each type of DD. Differences in percentages were evaluated using Rao-Scott chi-square tests with p*<*0.05 considered statistically significant. To reflect the complex sampling design and generate nationally representative estimates, all analyses accounted for clustering, stratification, and weights, using SAS software (version 9.4; SAS Institute) and were verified using SUDAAN (version 11.0.1; RTI International).

Of the 44,866 children aged 3–17 years included in the 2014–2018 NHIS, 567 were excluded because of missing information on any question related to DDs or abilities, health needs, or use of services, resulting in a total of 44,299. The estimated prevalence of DDs among U.S. children aged 3–17 years was 17.3%, ranging from 0.2% (blindness) to 9.4% (attention-deficit/hyperactivity disorder) (Table 1); 6.7% of U.S. children had two or more DDs. Among children with DDs, 5.7% had limited movement or play abilities, 4.7% needed help with personal care, 4.6% needed special equipment, and 2.4% received home health care, compared with ≤1% of children without DDs. Children with DDs were two to seven times as likely as those without DDs to have taken prescription medication for ≥3 months (41.6% versus 8.4%), have seen a mental health professional (30.6% versus 4.5%), a medical specialist (26.0% versus 12.4%), or a therapist (25.0% versus 4.5%) during the past year (Table 1). Children with DDs were more likely to participate in special education or EIS (41.9% versus 2.4%). The percentage of children with limited abilities, special health needs, or who used specialty services was higher among children with two or more DDs than among those with one DD or none. Children with each type of DD were more likely than were those without DDs to have limited abilities or special health needs, or to use specialty services.

**TABLE 1:** Prevalence of developmental delays, disorders, or disabilities among children and adolescents aged 3–17 years and percentage with selected functional abilities, health needs, and related service use, by type and number of developmental delays, disorders, or disabilities — National Health Interview Survey, United States 2014–2018

DD	No.^†^	% (95% CI)*									
		Prevalence	Abilities	Special health needs				Specialty services used			
			Limited ability to crawl, walk, run, or play	Needs help with personal care	Needs special equipment	Received home health care	Took prescription medications for ≥3 months	Saw a mental health professional	Saw a medical specialist	Saw a therapist^§^	Receives special education or EIS
No DDs	36,582	82.7 (82.2– 83.2)	1.0 (0.8–1.1)	0.1 (0.1–0.1)	0.7 (0.6–0.8)	0.3 (0.3–0.4)	8.4 (8.0–8.7)	4.5 (4.2–4.8)	12.4 (11.9–12.9)	4.5 (4.2–4.8)	2.4 (2.2–2.6)
Any DDs^¶^	7,717	17.3 (16.8–17.8)	5.7 (5.0–6.4)	4.7 (4.1–5.4)	4.6 (4.0–5.3)	2.4 (1.9–2.9)	41.6 (40.1–43.1)	30.6 (29.2–32.0)	26.0 (24.7–27.3)	25.0 (23.7–26.3)	41.9 (40.5–43.4)
One DD	4,674	10.5** (10.2–10.9)	3.0 (2.4–3.8)	1.3 (0.9–1.9)	2.9 (2.3–3.6)	0.9 (0.6–1.3)	34.8 (33.0–36.7)	22.9 (21.2–24.6)	21.4 (19.8–23.0)	17.6 (16.1–19.2)	27.4 (25.6–29.2)
Two or more DDs	3,043	6.7** (6.4–7.0)	9.8 (8.4–11.3)	10.0 (8.6–11.6)	7.2 (6.0–8.6)	4.7 (3.7–5.9)	52.3 (49.9–54.7)	42.6 (40.3–45.0)	33.3 (30.9–35.7)	36.5 34.2–38.8)	64.8 (62.5–67.0)
ADHD	4,280	9.4 (9.0–9.8)	3.2 (2.5–3.9)	2.8 (2.1–3.7)	1.7 (1.3–2.3)	2.0 (1.4–2.7)	58.4 (56.3–60.5)	41.0 (39.0–43.0)	25.7 (23.9–27.5)	16.1 (14.7–17.6)	37.1 (35.2–39.1)
Autism spectrum disorder	1,064	2.4 (2.2–2.6)	9.7 (7.4–12.5)	17.3 (14.3–20.8)	4.8 (3.2–6.8)	6.7 (4.6–9.4)	44.2 (40.2–48.2)	49.9 (46.1–53.8)	34.5 (30.4–38.8)	45.4 (41.4–49.4)	73.1 (69.6–76.5)
Blindness	65	0.2 (0.1–0.2)	42.9 (28.0–58.8)	37.4 (22.9–53.7)	39.2 (25.1–54.8)	15.9^††^ (5.8–32.3)	38.9 (23.9–55.6)	10.4^††^ (3.7–21.9)	48.9 (33.1–64.9)	29.8 (18.2–43.8)	50.1 (35.0–65.3)
Cerebral palsy	114	0.3 (0.2–0.4)	71.7 (58.9–82.3)	40.1 (28.2–52.9)	55.7 (42.9–67.9)	15.4 (8.1–25.7)	50.5 (37.9–63.1)	29.5 (18.0–43.4)	72.1 (60.9–81.6)	69.5 (57.2–80.1)	68.5 (56.0–79.2)
Moderate-to-profound hearing loss	268	0.6 (0.5–0.7)	16.1 (10.1–23.8)	11.1 (6.7–17.0)	39.3 (31.6–47.4)	3.6^††^ (1.3–7.7)	33.1 (25.4–41.5)	25.2 (18.4–32.9)	39.5 (31.5–47.9)	48.0 (39.9–56.3)	40.2 (32.6–48.1)
Learning disability^§§^	2,941	6.5 (6.2–6.9)	5.2 (4.1–6.5)	4.6 (3.6–5.7)	3.5 (2.6–4.7)	2.6 (1.9–3.5)	39.8 (37.5–42.2)	34.3 (31.8–36.8)	25.4 (23.3–27.6)	29.7 (27.4–32.0)	60.6 (58.1–63.0)
Intellectual disability	529	1.1 (1.0–1.3)	24.8 (20.1–30.0)	30.8 (25.2–36.9)	18.7 (14.5–23.4)	9.9 (6.3–14.6)	53.7 (47.9–59.4)	43.7 (37.9–49.6)	47.7 (41.8–53.7)	49.3 (43.6–55.0)	81.7 (77.1–85.7)
Seizures	332	0.7 (0.6–0.9)	22.5 (16.4–29.6)	18.3 (12.5–25.3)	16.0 (10.6–22.8)	8.2 (4.8–12.9)	62.3 (53.6–70.4)	24.5 (18.2–31.7)	53.1 (45.6–60.5)	30.4 (24.1–37.4)	39.3 (32.0–47.0)
Stuttering	842	2.0 (1.8–2.2)	8.6 (6.4–11.3)	8.3 (5.9–11.2)	4.5 (3.0–6.4)	2.3 (1.2–3.9)	31.0 (27.1–35.0)	25.1 (21.4–29.1)	23.7 (20.2–27.4)	40.5 (36.1–45.0)	41.0 (36.6–45.4)
Other developmental delay	1,732	3.9 (3.6–4.1)	12.7 (10.5–15.2)	10.6 (8.7–12.6)	9.8 (7.8–12.0)	3.6 (2.6–4.9)	35.4 (32.4–38.4)	29.4 (26.6–32.3)	33.3 (30.3–36.4)	47.5 (44.4–50.6)	59.4 (56.3–62.5)

**Abbreviations:** ADHD = attention-deficit/hyperactivity disorder; DDs = developmental delays, disorders, or disabilities; EIS = early intervention services.

* Weighted estimates and 95% CIs account for the complex survey design.

^†^ Unweighted number of children; children might have more than one DD, except as indicated.

^§^ A physical therapist, speech therapist, respiratory therapist, audiologist, or occupational therapist.

^¶^ Children whose parents answered affirmatively to questions for one or more of the 10 conditions listed in the table, regardless of the number of conditions reported.

** Does not sum to percentage of children with any DD because of rounding.

^††^ Potentially unreliable estimates based on a relative standard error ≥30% and <50%.

^§§^ Children with both intellectual disability and learning disability were not included in the estimate of children with learning disability.

Among children with DDs, the percentage with limited abilities and special health needs, and who used specialty services varied across sociodemographic subgroups (Table 2). Compared with non-Hispanic White children with DDs, a lower percentage of non-Hispanic Black, non-Hispanic other, and Hispanic children with DDs took prescription medication. Compared with non-Hispanic White children with DDs, Hispanic children with DDs were less likely to have seen a mental health professional, and non-Hispanic Black and Hispanic children with DDs were less likely to have seen a medical specialist. Compared with children aged 3–8 years who had DDs, a lower percentage of children aged 9–17 years with DDs needed special equipment or help with personal care, received home health care, or saw a therapist, whereas a higher percentage took prescription medications or saw a mental health professional.

**TABLE 2:** Prevalence of selected functional disabilities, health needs, and related service use among U.S. children and adolescents aged 3–17 years with one or more developmental delays, disorders, or disabilities,* by socioeconomic and demographic group — National Health Interview Survey, United States, 2014–2018

Demographic group	No.^§^	% (95% CI)^†^								
		Abilities	Special health needs				Specialty services used			
		Limited ability to crawl, walk, run, or play	Needs help with personal care	Needs special equipment	Received home health care	Took prescription medications for ≥3 months	Saw a mental health professional	Saw a medical specialist	Saw a therapist^¶^	Receives special education or EIS
**Sex**										
Male	5,071	4.9 (4.1–5.8)	4.5 (3.7–5.4)	3.8 (3.1–4.6)	2.1 (1.6–2.7)	42.5 (40.6–44.3)	31.5 (29.8–33.3)	25.5 (23.8–27.2)	25.8 (24.1–27.5)	43.2 (41.4–45.0)
Female	2,646	7.1 (5.8–8.6)	5.1 (4.1–6.4)	6.0 (4.9–7.3)	2.8 (2.0–3.9)	40.0 (37.7–42.5)	28.8 (26.5–31.1)	27.0 (24.8–29.3)	23.5 (21.3–25.9)	39.6 (37.1–42.1)
p*-*value**	NA	0.004	0.357	0.001	0.154	0.109	0.052	0.282	0.130	0.020
**Race or ethnicity**										
Black, non-Hispanic	1,083	5.4 (3.4–8.1)	5.1 (3.1–7.7)	4.6 (3.0–6.8)	2.4^††^ (1.1–4.5)	40.0^§§^ (36.2–43.9)	29.4 (26.0–33.0)	22.0^§§^ (18.7–25.5)	23.3 (19.9–27.1)	43.4 (39.5–47.4)
White, non-Hispanic	4,398	5.4 (4.5–6.3)	4.1 (3.4–5.0)	4.7 (3.9–5.6)	2.1 (1.6–2.8)	46.3 (44.4–48.3)	32.0 (30.2–33.9)	28.4 (26.6–30.2)	24.3 (22.6–26.1)	40.9 (38.9–42.9)
Other, non-Hispanic	653	5.4 (3.4–8.2)	4.7 (2.7–7.5)	5.1 (3.1–7.7)	2.3 (1.2–3.9)	38.1^§§^ (33.1–43.2)	34.9 (29.7–40.3)	24.5 (20.5–28.8)	24.4 (20.2–29.0)	40.9 (35.8–46.1)
Hispanic	1,570	6.7 (5.1–8.6)	6.0 (4.5–7.8)	4.1 (2.9–5.5)	2.9 (1.9–4.4)	31.0^§§^ (28.1–34.0)	26.1^§§^ (23.1–29.1)	23.2^§§^ (20.7–25.9)	27.8 (24.6–31.3)	44.0 (40.6–47.3)
p*-*value**	NA	0.549	0.213	0.842	0.662^††^	<0.001	0.002	<0.001	0.171	0.344
**Age group, yrs**										
3–8	2,127	6.8 (5.4–8.4)	7.3 (5.9–8.9)	5.9 (4.6–7.4)	3.0 (2.2–4.1)	32.9 (30.3–35.6)	26.8 (24.3–29.4)	27.7 (25.3–30.2)	42.6 (39.9–45.3)	44.4 (41.7–47.1)
9–11	1,673	4.8 (3.4–6.6)	5.4 (3.9–7.4)	3.9^¶¶^ (2.8–5.2)	3.5 (2.2–5.3)	46.8^¶¶^ (43.6–50.1)	33.3^¶¶^ (30.3–36.4)	23.5 (20.8–26.3)	23.3^¶¶^ (20.6–26.2)	42.1 (39.0–45.3)
12–17	3,917	5.4 (4.5–6.4)	2.8^¶¶^ (2.2–3.5)	4.1^¶¶^ (3.3–5.1)	1.4^¶¶^ (1.0–1.9)	44.5^¶¶^ (42.3–46.7)	31.6^¶¶^ (29.7–33.6)	26.1 (24.3–28.0)	14.9^¶¶^ (13.4–16.6)	40.3 (38.2–42.5)
p-value**	NA	0.133	<0.001	0.029	0.001	<0.001	0.001	0.061	<0.001	0.063
**Mother’s education**										
Less than HS or GED	2,375	6.0 (4.8–7.5)	5.1 (4.0–6.5)	4.5 (3.4–5.7)	2.2 (1.4–3.1)	35.9 (33.5–38.3)	25.9 (23.7–28.3)	22.3 (20.0–24.8)	24.9 (22.6–27.4)	45.4 (42.7–48.0)
HS or greater	4,461	5.6 (4.7–6.7)	4.7 (3.9–5.7)	4.9 (4.0–5.8)	2.6 (2.0–3.4)	43.8 (41.7–45.8)	32.4 (30.5–34.3)	28.9 (27.1–30.7)	25.8 (24.1–27.7)	40.5 (38.6–42.4)
p*-*value**	NA	0.613	0.575	0.577	0.422	<0.001	<0.001	<0.001	0.552	0.003
Poverty status***										
<100% FPL	1,605	6.3 (4.8–8.2)	4.9 (3.5–6.7)	5.1 (3.6–6.9)	2.4 (1.5–3.6)	40.5 (37.3–43.7)	32.2 (29.1–35.3)	23.0 (20.4–25.8)	24.4 (21.6–27.4)	45.1 (41.8–48.5)
≥100% FPL	5,885	5.3 (4.6–6.2)	4.6 (3.9–5.4)	4.4 (3.7–5.2)	2.4 (1.8–3.0)	42.4 (40.7–44.1)	30.2 (28.6–31.9)	27.4 (25.8–29.0)	25.1 (23.5–26.6)	41.0 (39.3–42.8)
p*-*value**	NA	0.250	0.697	0.427	0.916	0.294	0.256	0.006	0.695	0.035
**U.S. Census Bureau region of residence** ^†††^										
Northeast	1,314	6.7 (4.7–9.1)	6.0^§§§^ (4.2–8.3)	5.6 (3.9–7.8)	4.3^§§§^ (2.7–6.4)	40.5 (36.6–44.6)	36.3^§§§^ (33.1–39.5)	30.3^§§§^ (26.9–34.0)	29.6^§§§^ (26.0–33.4)	54.3^§§§^ (50.5–58.0)
South	2,946	5.1 (4.1–6.3)	3.6 (2.7–4.6)	3.8 (2.9–4.9)	1.4 (1.0–2.0)	45.1 (42.8–47.4)	26.6 (24.3–29.0)	24.7 (22.7–26.9)	21.5 (19.5–23.5)	37.2 (34.9–39.6)
Midwest	1,688	5.0 (3.8–6.4)	4.3 (3.2–5.6)	5.2 (4.0–6.7)	2.4 (1.5–3.5)	43.5 (40.3–46.7)	31.1^§§§^ (28.2–34.1)	26.2 (23.5–29.2)	26.1^§§§^ (23.3–29.1)	39.5 (36.5–42.6)
West	1,769	6.8 (5.1–8.8)	6.3^§§§^ (4.8–8.2)	4.6 (3.3–6.0)	2.6^§§§^ (1.7–3.8)	33.3^§§§^ (30.3–36.3)	33.0^§§§^ (29.9–36.3)	24.5 (21.8–27.3)	26.7^§§§^ (23.9–29.6)	43.7^§§§^ (40.7–46.7)
p*-*value**	NA	0.196	0.007	0.168	0.001	<0.001	<0.001	0.023	<0.001	<0.001
**Health insurance** ^¶¶¶^										
None	342	3.1^††^ (1.3–6.0)	2.6^††^ (0.9–5.8)	3.1^††^ (1.3–6.1)	—****	21.4 (16.2–27.2)	19.5 (14.0–26.2)	11.3 (7.7–15.8)	14.9 (9.9–21.3)	31.4 (24.5–39.0)
Any	7,351	5.8 (5.1–6.6)	4.8 (4.2–5.5)	4.7 (4.0–5.4)	2.4 (2.0–3.0)	42.6 (41.1–44.2)	31.0 (29.6–32.5)	26.7 (25.3–28.1)	25.4 (24.1–26.8)	42.4 (40.9–43.9)
p*-*value**	NA	0.066	0.145	0.249	—****	<0.001	0.001	<0.001	0.002	0.004

**Abbreviations:** DDs = developmental delays, disorders, or disabilities; EIS = early intervention services; FPL = federal poverty level; GED = general educational development certificate; HS = high school; NA = not applicable.

* Children whose parents answered affirmatively to questions for one or more of 10 selected conditions (attention-deficit/hyperactivity disorder, autism spectrum disorder, blindness, cerebral palsy, moderate to profound hearing loss, learning disability, intellectual disability, seizures, stuttering, or other DD), irrespective of the number of conditions reported (7,717).

^†^ Weighted estimates and 95% CIs account for the complex survey design.

^§^ The unweighted number of children with any DD in the specified demographic group.

^¶^ A physical therapist, speech therapist, respiratory therapist, audiologist, or occupational therapist.

** p*-*value for Rao-Scott chi-square test for difference in percentages among subgroups.

^††^ Potentially unreliable estimates based on a relative standard error ≥30% and <50%, or comparison based on a group with an unreliable estimate.

^§§^ Significantly different from non-Hispanic White children (p<0.05; Rao-Scott chi-square test).

^¶¶^ Significantly different from children aged 3–8 years (p<0.05; Rao-Scott chi-square test).

*** Ratio of family income to FPL.

^†††^ *Midwest:* Kansas, Illinois, Indiana, Iowa, Michigan, Minnesota, Missouri, Nebraska, North Dakota, Ohio, South Dakota, and Wisconsin; *Northeast:* Connecticut, Maine, Massachusetts, New Hampshire, New Jersey, New York, Pennsylvania, Rhode Island, and Vermont; *South:* Alabama, Arkansas, Delaware, District of Columbia, Florida, Georgia, Kentucky, Louisiana, Maryland, Mississippi, North Carolina, Oklahoma, South Carolina, Tennessee, Texas, Virginia, and West Virginia; and *West:* Alaska, Arizona, California, Colorado, Hawaii, Idaho, Montana, Nevada, New Mexico, Oregon, Utah, Washington, and Wyoming.

^§§§^ Significantly different from children living in the South (p<0.05; Rao-Scott chi-square test).

^¶¶¶^ Any health insurance coverage at the time of the interview under private health insurance. Medicare, Medicaid, State Children’s Health Insurance Program, a state-sponsored health plan, other government programs, or military health plan (includes TRICARE, Veterans Affairs, and Civilian Health and Medical Program of the Department of Veterans Affairs).

**** Unreliable estimate based on a relative standard error ≥50% or comparison based on a group with an unreliable estimate are not shown.

Among children with DDs, those whose mother had less than a high school education were less likely to take prescription medication or to see specialty health care professionals, but more likely to receive special education or EIS. Compared with children living above the federal poverty level, those living at or below the federal poverty level were less likely to see a medical specialist and more likely to receive special education or EIS. The percentage of children with DDs who needed help with personal care or received home health care and used services was higher in the Northeast and West than in the South; a higher percentage in the Midwest saw a mental health professional or therapist, and a lower percentage in the West took prescription medications for ≥3 months. The percentage of children with DDs who took prescription medication for ≥3 months, saw medical specialists, or received special education or EIS services was lower among those without health insurance.

## Discussion

During 2014–2018, approximately one in six (17.3%) children had a DD, and one in 15 (6.7%) had two or more DDs. Children with DDs have a higher prevalence of limited ability to move or play, health needs, and specialized service use compared to those without DDs. The prevalences of DDs during 2014–2018, overall and by type, are consistent with 2015–2017 (*1*). Although differing DD definitions and study methods used in previous years present challenges to comparing the findings in this report with data from 1997–2005 and 2006–2010, the percentage of U.S. children with any DD who had limitations in movement or play appeared to be slightly lower during 2014–2018 overall, but not for children with blindness, cerebral palsy, or hearing loss (*2*,*3*). In contrast, the percentage of U.S. children with special health needs or who took prescription medications, saw specialty health care providers, or received education services appeared to be similar or higher during 2014–2018 than 1997–2005, with the exception of children with autism spectrum disorder (*2*,*3*). One explanation for potential decreases in health needs and service use over time is the inclusion of children with less significant support needs associated with autism spectrum disorder.**

This study provides new data on sociodemographic differences in the health needs and use of special services among children with DDs. The observed differences could be associated with differential access to care resulting from a variety of factors, including health insurance coverage, specialist proximity, language or cultural barriers, and variability in practices and policies (*4*,*6*–*8*). A 2018 study examining health care coverage and access among children, adolescents, and young adults during 2010–2016 suggests that significant improvements in health care coverage occurred with the implementation of the Affordable Care Act in 2010, yet gaps remain, particularly among adolescents as they transition to adult care (*7*). Eligibility criteria, service availability, long waiting times, cost, and lack of information are reported barriers to receipt of services for children with DDs (*8*). Referral practices and coordination across early childhood service providers and systems also affect access to early intervention for young children (*4*,*6*). Lower service use associated with poverty is of concern given the impact of poverty on child development (*4*,*7*–*9*). Although implementation of programs in low-income settings might help increase early identification of DDs among children living in poverty, one study of treatment for children with autism spectrum disorder suggests that differential service by geographic region might not be explained by child and family characteristics (*10*). In addition, lower service use associated with race or ethnic identification is of concern given the pervasive impact of racism on child development (*4*,*7*–*9*). More work is needed to ensure that children with identified delays receive a diagnosis and services through enhanced access, coordination of care across systems (e.g., school, health care, and community), and increased workforce capacity (*7*–*9*). Strategies and programs that support families, health care, education, and social service providers with evidence-based interventions and tools to promote early identification and coordinated care across systems for children with DDs could potentially improve access to needed health care and services.^††^

The findings in this report are subject to at least four limitations. First, information is reported by the parent and has not been independently verified; therefore, it might be subject to recall bias or variation in interpretation. Second, the reported DDs in this analysis are a heterogeneous grouping that vary materially in severity, prevalence, and persistence over time. Third, children’s symptoms and abilities relevant to diagnosis or their eligibility for services might change with intervention or age. Finally, estimates are unadjusted for demographic or other characteristics; thus, observed differences across groups might be attributable to other factors, such as other medical conditions or contextual factors.

These data confirm that DDs are common and often co-occur, and that children with DDs have more health-related needs and service use than do children without DDs. Strategies that promote early identification and coordination of services for children with DDs could improve health and reduce the need for services later in life. Inequities in use and receipt of medications and services by sociodemographic subgroups deserve further investigation to guide development and implementation of strategies to promote health equity and ensure that all children with DDs have access to needed care and services to enable them to thrive.

**Summary**

**What is already known about this topic?**

Developmental delays, disorders, and disabilities (DDs) are common among U.S. children and adolescents.

**What is added by this report?**

Approximately one in six (17.3%) U.S. children and adolescents aged 3–17 years had DDs during 2014–2018. Compared with children and adolescents without DDs, those with DDs were two to seven times as likely to take prescription medication and receive mental health or specialized health care provider services and 18 times as likely to receive special education or early intervention services.

**What are the implications for public health practice?**

Policies and programs that promote early identification of children and adolescents with DDs and increase access to intervention services could improve health and reduce the need for services later in life.

## Footnotes

*
https://acl.gov/sites/default/files/about-acl/2016-12/dd_act_2000.pdf

† Attention-deficit/hyperactivity disorder, autism spectrum disorder, blindness, cerebral palsy, moderate-to-profound hearing loss, learning disability, intellectual disability, seizures in the past 12 months, stutter or stammering in the past 12 months, or any other developmental delay.

§
https://sites.ed.gov/idea/statute-chapter-33/subchapter-iii/1431

¶
https://www.cdc.gov/nchs/nhis/index.htm

**
https://pubmed.ncbi.nlm.nih.gov/26632847/

†† CDC’s Learn the Signs. Act Early. (https://www.cdc.gov/ActEarly); CDC-funded Association of University Centers on Disabilities Children’s Mental Health Champions (https://nationalcenterdph.org/our-focus-areas/wellness-and-mental-health/mental-health-champions/); CDC-funded programs through the National Resource Center on ADHD resources (https://www.cdc.gov/ADHD) and the Tourette Association of America (https://www.cdc.gov/Tourette); CDC’s epilepsy program (https://www.cdc.gov/epilepsy/groups/parents.htm); Department of Education, Office of Special Education Program’s Parent Centers (https://www.parentcenterhub.org) and the Health Resources Services Administration, Maternal and Child Health Bureau’s Early Childhood Comprehensive Systems (https://mchb.hrsa.gov/earlychildhoodcomprehensivesystems); Healthy Start (https://mchb.hrsa.gov/maternal-child-health-initiatives/healthy-start); Home Visiting (https://mchb.hrsa.gov/maternal-child-health-initiatives/home-visiting-overview); Leadership Education in Neurodevelopmental and Other Related Disabilities (LEND) program (https://mchb.hrsa.gov/training/projects.asp?program); and Got Transition (https://www.hrsa.gov/library/got-transition).
